# Next-Generation Sequencing Reveals Novel Genetic Variants (SRY, DMRT1, NR5A1, DHH, DHX37) in Adults With 46,XY DSD

**DOI:** 10.1210/js.2019-00306

**Published:** 2019-10-10

**Authors:** Federica Buonocore, Oliver Clifford-Mobley, Tom F J King, Niccolò Striglioni, Elim Man, Jenifer P Suntharalingham, Ignacio del Valle, Lin Lin, Carlos F Lagos, Gill Rumsby, Gerard S Conway, John C Achermann

**Affiliations:** 1 Genetics and Genomic Medicine, UCL Great Ormond Street Institute of Child Health, University College London, London, United Kingdom; 2 Clinical Biochemistry, University College London Hospitals, London, United Kingdom; 3 Reproductive Medicine Unit, University College London Hospitals, London, United Kingdom; 4 Chemical Biology and Drug Discovery Laboratory, Facultad de Medicina y Ciencia, Universidad San Sebastián, Santiago, Chile

**Keywords:** desert hedgehog, DSD, DHX37, sex determination, SRY, steroidogenic factor-1

## Abstract

**Context:**

The genetic basis of human sex development is slowly being elucidated, and >40 different genetic causes of differences (or disorders) of sex development (DSDs) have now been reported. However, reaching a specific diagnosis using traditional approaches can be difficult, especially in adults where limited biochemical data may be available.

**Objective:**

We used a targeted next-generation sequencing approach to analyze known and candidate genes for DSDs in individuals with no specific molecular diagnosis.

**Participants and Design:**

We studied 52 adult 46,XY women attending a single-center adult service, who were part of a larger cohort of 400 individuals. Classic conditions such as17*β*-hydroxysteroid dehydrogenase deficiency type 3, 5*α*-reductase deficiency type 2, and androgen insensitivity syndrome were excluded. The study cohort had broad working diagnoses of complete gonadal dysgenesis (CGD) (n = 27) and partially virilized 46,XY DSD (pvDSD) (n = 25), a group that included partial gonadal dysgenesis and those with a broad “partial androgen insensitivity syndrome” label. Targeted sequencing of 180 genes was undertaken.

**Results:**

Overall, a likely genetic cause was found in 16 of 52 (30.8%) individuals (22.2% CGD, 40.0% pvDSD). Pathogenic variants were found in sex-determining region Y (SRY; n = 3), doublesex and mab-3–related transcription factor 1 (DMRT1; n = 1), NR5A1/steroidogenic factor-1 (SF-1) (n = 1), and desert hedgehog (DHH; n = 1) in the CGD group, and in NR5A1 (n = 5), DHH (n = 1), and DEAH-box helicase 37 (DHX37; n = 4) in the pvDSD group.

**Conclusions:**

Reaching a specific diagnosis can have clinical implications and provides insight into the role of these proteins in sex development. Next-generation sequencing approaches are invaluable, especially in adult populations or where diagnostic biochemistry is not possible.

It is now more than 25 years since SRY was identified as the main testis-determining gene in humans and mice and as a cause of 46,XY differences (disorders) of sex development (DSDs) [1–3]. Since then, at least 40 other genetic causes of DSDs have been reported, but the relative contribution to these genes within the clinical setting is poorly documented [4–6].

DSDs are usually considered in three broad categories: sex chromosome DSD, 46,XY DSD, and 46,XX DSD [7]. Although the diagnosis of specific forms of sex chromosome DSD and 46,XX DSD can usually be made with karyotyping or biochemical analysis (for congenital adrenal hyperplasia), the specific diagnosis of 46,XY DSD is often more challenging.

46,XY DSD is commonly divided into conditions affecting gonad (testis) development (*e.g.*, complete gonadal dysgenesis, also known as Swyer syndrome) or conditions affecting androgen biosynthesis and action. Proximal genetic blocks in androgen biosynthesis are rare and usually affect the adrenal gland as well as the gonad (steroidogenic acute regulatory protein/*STAR*), P450 side-chain cleavage/*CYP11A1*, 3*β*-hydroxysteroid dehydrogenase type 3/*HSD3B2*, 17*α*-hydroxylase/*CYP17A1*, P450 oxidoreductase/*POR*). More specific defects in testosterone biosynthesis [17*β*-hydroxysteroid dehydrogenase deficiency (17*β*-HSD) type 3 (*HSD17B3*)], in the conversion of testosterone to dihydrotestosterone [5*α*-reductase deficiency type 2 (*SRD5A2*)], or in androgen action [androgen insensitivity syndrome (AIS), androgen receptor] can sometimes be suspected on clinical or biochemical grounds, but genetic testing is becoming increasingly important given the overlap in clinical features among various causes [8, 9].

To complicate diagnosis further, each condition can have a spectrum of genital phenotypes depending on the underlying variant and other modifying factors. This situation is especially apparent for changes in steroidogenic factor-1 (SF-1; encoded by *NR5A1*), where clinical and endocrine features ranging from complete gonadal dysgenesis through to hypospadias or male factor infertility can be seen [10, 11].

Newer technologies such as high-throughput next-generation sequencing (NGS) now allow the analysis of many genes simultaneously in the DNA from one individual. Panel-based approaches and exome sequencing have been reported recently for the diagnosis of several endocrine conditions such as neonatal diabetes mellitus and primary adrenal insufficiency [12, 13], as well as for DSDs [14–20].

In this study, we report the results of a targeted NGS analysis of a relatively large cohort of individuals with 46,XY DSD who had the working diagnosis of complete gonadal dysgenesis (CGD) (n = 27) or what we term “partially virilized 46,XY DSD” (pvDSD) (n = 25), a group that included partial gonadal dysgenesis (PGD) and those with a broad partial androgen insensitivity syndrome (PAIS) label (androgen receptor negative). We analyzed >150 genes that are either established or potential causes of 46,XY DSD, or candidate genes for 46,XY DSD based on our published “atlas” of differential transcriptomic expression during early human embryonic/fetal gonad development [21].

## 1. Materials and Methods

### A. Study Cohort

**Figure 1.**
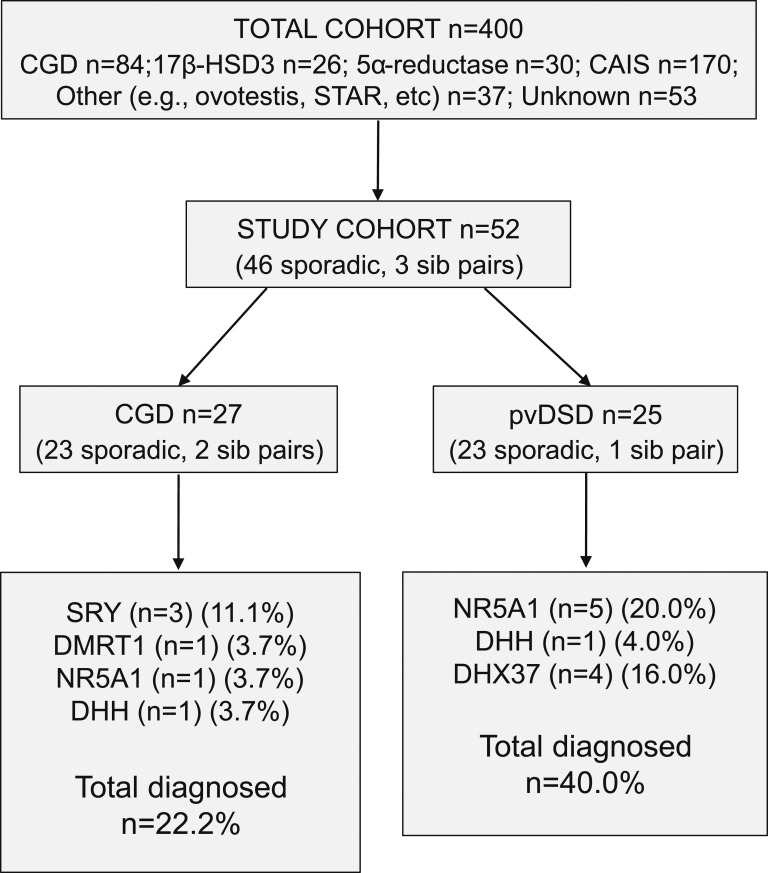
Overview of the study cohort and summary of findings.

The study was undertaken in a cohort of 52 individuals (46 sporadic, 3 sibling pairs) with 46,XY DSD of unknown molecular etiology attending adult DSD clinics at the University College London Hospitals NHS Foundation Trust (Fig. 1). This adult DSD clinic takes place in the setting of a Department of Women’s Health. Therefore, the spectrum of DSD phenotype is predominantly female. Consecutive subjects were approached without clinical selection criteria providing a real-life clinical cohort. These individuals were part of a larger cohort of 400 women with 46,XY DSD, where 17*β*-HSD type 3 (*HSD17B3*) (n = 26), 5*α*-reductase deficiency (5ARD) type 2 (*SRD5A2*) (n = 30), and AIS, androgen receptor (n = 170) had been prescreened (Fig. 1) [5, 6].

A total of 27 women had a working diagnosis of CGD and mostly presented with female-typical genitalia and primary amenorrhea/absent puberty in adolescence (Swyer syndrome). They were recruited from a total cohort of 84 women with CGD.

Another 25 individuals presented with atypical genitalia and were brought up as girls with a working diagnosis of PGD or PAIS. They were recruited from a total of 53 women with this phenotype where the underlying diagnosis was unknown. Historically, many individuals have been labeled under the “umbrella” term PAIS, without genetic or biochemical evidence of the diagnosis. We have termed this group pvDSD.

Individuals with adrenal insufficiency or high steroidogenic blocks/congenital adrenal hyperplasia, renal anomalies, or obvious syndromic associations of DSD were excluded.

Informed consent was given by all participants. Research Ethics Committee approval was obtained as part of the Reproductive Life Course Project (IRAS project ID 184646, REC reference 16/LO/0682) or study of the genetics of reproductive biology (07Q050824).

### B. Genetic Analysis

DNA was extracted from peripheral blood leukocytes using standard methods and subjected to genetic analysis using the approaches outlined below.

#### B-1. Sanger sequencing for *NR5A1*

Analysis of *NR5A1* was undertaken using standard Sanger sequencing with primers and conditions reported previously [22].

#### B-2. HaloPlex^®^ targeted NGS panel

A HaloPlex DNA targeted gene enrichment panel (Agilent Technologies, Santa Clara, CA) was designed using SureDesign software (www.agilent.com/genomics/suredesign). This panel included 168 known DSD-associated genes as well as potential candidate genes (Table 1). The panel captured all coding exons and 100 bp of intronic flanking sequence of genes of interest with predicted target coverage >98.87%.

**Table 1.** Summary of Genes Included on the Targeted Panel

Genes implicated in DSD (n = 53)
*SRY, SOX9, NR5A1, WT1, NR0B1, LHCGR, STAR, CYP11A1, HSD3B2, CYP17A1, POR, HSD17B3, SRD5A2, AR, CYP19A1, CDKN1C, SAMD9*
*CBX2, MAP3K1, GATA4, MAMLD1, HHAT, DHH, SOX8, CYB5A, AKR1C2, AKR1C4, SOX3, RSPO1, ARX, DHCR7, AMH, AMHR2, POU1F1, DHX37, MYRF, DMRT1, FGF9, FGFR2, WWOX, ANOS1, CHD7, ESR2, FGF8, FGFR1, PROK2, PROKR2, STARD8, WDR11, ZFPM2, ZNRF3, PBX1 TSPYL1*
**Other genes sequenced (n = 127)**
*WNT4, CXCL12, MAP3K15, MAPK15, MAPK4, FOXO4, ABCA10, ABCA9, ACAT2, ADAMTS5, AGPAT9, ALAS1, ALDH1A1, AMIGO2, ANKRD18A, AOC3, APOA1, APOBEC3G, ARAP2, ASPN,, BNC1, BNC2, BRDT, CALB2, CARTPT, DHCR24, CD96, CD99, CILP, CITED1, CITED4, CNTNAP4, CORO2A, CTSV, CUL4B, EGFLAM, ELOVL2, ENTPD3, EPPIN, FATE1, FDX1, FDXR, RMPD1, G6PD, GLI4, GNRHR, GPD1L, GPX1, GRAMD1B, GSTA1, HIST1H2AA, HK2, HOXA10, HPGD, HPRT1, HRASLS5, HSD17B11, HSD17B6, HSD3B1, IDI1, IFI16, ILDR2, INHA, INHBB, INHBE, INSL3, ITGA9, JAM2, KEL, LDLR, MAGEB1, MANEA, MGARP, MOCOS, MRO, MSC, NANOG, NLRP7, NOSTRIN, NOTCH2, NPY, NPY1R, NRK, OPN3, OR10G9, OR4D5, OR8D4, PEG3, PHF24, PLEKHB1, PRND, PRPS2, PTCH1, PTCH2, RASSF2, REEP1, RELN, REN, RGAG1, RMDN2, SCARB1, SCUBE1, SERPINA5, SERPINF1, SESN1, SHH, SLAIN1, SLC16A9, SLC27A3, SLC52A3, SLC8B1, SMOC1, SOAT1, SORCS1, SRA1, SSFA2, STARD4, STK10, SUSD3, TCF21, TFAP2C, TMC5, TSPYL2, VCAM1, ZFAND5, ZNF280B, ZNF676*

Underlined genes were sequenced using a Nonacus panel.

Genomic DNA samples (225 ng) were processed for Illumina sequencing according to the HaloPlex target enrichment system protocol (version D.5, Agilent Technologies) and as described previously [13]. Resultant libraries were then subjected to NGS using an Illumina MiSeq platform (Illumina, San Diego, CA). Raw FASTQ files were analyzed using SureCall (version 3.0.1.4) software (Agilent Technologies). Visual inspection of BAM files was also undertaken to ensure that deletions of key genes were not overlooked.

#### B-3. Nonacus^®^ targeted NGS panel

A further 12 potential known and candidate genes for DSD were analyzed using a Cell3 Target enrichment panel for NGS (Nonacus, Birmingham, UK) (Table 1). This captured all coding exons, including a 100-bp intronic flanking sequence, with a predicted target coverage of 97.35%.

Genomic DNA samples (100 ng) were prepared for Illumina sequencing according to Cell3™ Target, a cell-free DNA target enrichment system for NGS (Illumina sequencers; version 1.2.2 protocol). In brief, DNA was sheared by an enzymatic fragmentation before undergoing end repair and dA tailing. This enables the Illumina unique molecular identifier adapters to be ligated on both 5′ and 3′ ends. DNA was then purified using Agencourt AMPure beads to remove residual nonligated adapters. DNA was amplified using primers that bind to the ligated adapter. Libraries were hybridized with the customized probes before undergoing a further bead wash to remove nontargeted DNA. Targeted library DNA sequences were then amplified using primers binding specifically to sequences within the Illumina adapters. A final bead clean-up step was carried out before quantification of captured libraries. These were then sequenced on a MiSeq platform (Illumina). Raw FASTQ files were analyzed using a bioinformatic pipeline provided by Nonacus, and consequent variant analysis was carried out in Ingenuity Variant Analysis software (Qiagen, Valencia, CA). Inspection of BAM files was carried out using the Integrative Genomics Viewer [23].

#### B-4. Variant validation

Validation of key variants identified was performed using Sanger sequencing. Regions of interest were PCR amplified and sequenced using a BigDye Terminator version 1.1 cycle sequencing kit (Applied Biosystems, Foster City, CA) on an ABI 3130 sequencer (Applied Biosystems), and visualized using Sequencher version 5.2.4 (Gene Codes Corporation, Ann Arbor, MI). Control data for population genomic variation was obtained using the Genome Aggregation Database (gnomAD) (gnomAD, Cambridge, MA, https://gnomad.broadinstitute.org) [24].

#### B-5. Functional prediction of genetic variants

Functional predictions of the effects of specific variants was performed using SIFT (http://provean.jcvi.org/index.php), PolyPhen2 (http://genetics.bwh.harvard.edu/pph2/), and MutationTaster algorithms (http://www.mutationtaster.org/). Conservancy mapping was undertaken with UniProt (https://www.uniprot.org). Depictions of protein domains were constructed using Domain Graph version 2.0 [25].

### C. Molecular Modeling and Simulation of DMRT–DNA Complexes

The molecular models of wild-type (WT) and mutant doublesex and mab-3–related transcription factor 1 (DMRT1) proteins were constructed using MODELLER, within the protein modeling module of Discovery Studio version 2.1 (Accelrys, San Diego, CA) [26]. The human DMRT1 sequence retrieved from the UniProt database (https://www.uniprot.org/uniprot/Q9Y5R6) was used as a reference, and only residues 70 to 130 were considered for modeling purposes [27]. WT and a p.R80S mutant were modeled using the recently reported crystal structure of human DMRT1 (residues 70 to 130) in complex with DNA (PDB ID 4YJ0) as a template [28].

For each protein model, a set of 100 models was constructed and the best one according to the MODELLER internal PDF score was selected for simulations steps. The DMRT1 WT and p.R80S mutant in complex with DNA systems were generated using VMD version 1.93, by inserting each protein into a 110- × 70- × 80-Å box consisting of a classic TIP3P model for water molecules, neutralized with Na^+^ or Cl^−^ ions [29, 30]. Periodic boundary conditions were imposed in all three directions, and the particle mesh Ewald method was used to account for full long-range electrostatic interactions within the selected boundary condition within a relative tolerance of 1 × 10^−6^ [31]. The final systems were composed of nearly 60,120 atoms.

Molecular dynamics simulations were carried out with the NAMD version 2.13 simulation package, using the CHARMM36 force field parameters for proteins [32–34]. The simulation was started with a brief energy minimization for 5000 steps, followed by 1 ns of heating with protein backbone sequential release of alpha carbon restraints (force constants were gradually reduced from 10 kcal/mol Å^2^ to 0 kcal/mol Å^2^) and 4 ns of equilibration, and 10 ns of production simulation for each protein was performed. The particle mesh Ewald method was used for full long-range electrostatics within a relative tolerance of 1 × 10^−6^. A 12-Å cutoff was used to compute nonbonded interactions with a smooth switching function applied at a distance of 10 Å. To impose the thermal exchange with an external thermostat, the isobaric-isothermal ensemble with a constant number of particles, pressure, and temperature was used. Constant temperature was maintained by coupling the system to a thermal bath whose temperature is maintained via Langevin dynamics with a friction coefficient of 1 ps^−1^. Constant pressure was maintained using a Langevin piston at a nominal value of 1 atm [35]. The SHAKE algorithm, with a tolerance of 1 × 10^−8^ Å, was applied to constrain the length of all covalent bonds involving hydrogen, thus allowing the use of a 2 fs integration time. Trajectory analyses and measurements were performed using VMD version 1.9.3 [29]. By plotting C*α*-root-mean-squared deviation along the molecular dynamic simulation, we assessed the structural equilibration reached by the models and the distances between the terminal heavy atom (Cz in Arg80 or O in the case of Ser80) and the nearest oxygen on the phosphate backbone. Figures were rendered using PyMol version 2.3.2 (Schrödinger, New York, NY; https://www.schrodinger.com).

### D. Transient Gene Transcription Assays

Expression vectors containing the NR5A1/SF-1 p.G22D or p.L420P missense variants were generated by site-directed mutagenesis (QuikChange, Stratagene, Amsterdam, Netherlands) using a WT pCMX-NR5A1 template and validated by direct sequencing. Transient transfection studies were performed using Lipofectamine 2000 (Invitrogen, Paisley, UK) in 96-well plates and a Dual-Luciferase reporter assay system (Promega, Madison, WI), as reported previously [22]. Studies were performed in tsa201 human embryonic kidney cells by transfecting empty, WT, or mutant NR5A1/SF-1 expression vectors (2  ng per well; p.G22D, p.L420P) with the SF-1–responsive minimal promoter of Cyp11a linked to luciferase (100  ng per well). Twenty-four hours following transfection, cells were lysed and luciferase activity was assayed (Dual-Luciferase reporter assay system, Promega; FLUOstar Optima, BMG Labtech, Aylesbury, UK), with standardization for *Renilla* coexpression. Results are shown as the mean ± SEM of three independent experiments, each performed in triplicate.

### E. Immunohistochemistry

Immunohistochemistry (IHC) for desert hedgehog (DHH) was undertaken in human fetal testis (9 weeks postconception) with ethical approval (REC reference 08/H0712/34+5) and informed consent in collaboration with the Human Developmental Biology Resource (http://www.hdbr.org). In brief, 12-µm sections were fixed briefly in 4% paraformaldehyde in Tris-buffered saline (TBS), rinsed in TBS, and blocked in 1% BSA in TBS with Tween 20 (0.5% Tween 20) before incubating overnight with mouse monoclonal anti-human DHH antibody (Santa Cruz Biotechnology, Santa Cruz, CA, sc-133244, 1:50 dilution) [36] and rabbit anti-human anti-Müllerian hormone antibody (Abcam, Cambridge, UK, ab-103233; 1:200 dilution) [37]. Sections were washed with TBS with Tween 20 (0.5% Tween 20) and then incubated for 1 hour with Alexa Fluor 488 goat anti-mouse (Invitrogen, A11001; 1:400) [38] and Alexa Fluor 555 goat anti-rabbit (Invitrogen, Waltham, MA, A21429; 1:400) [39], respectively, and counterstained with 4′,6-diamidino-2-phenylindole (Sigma-Aldrich, St. Louis, MO). Images were captured on a Zeiss LSM 710 confocal microscope (Carl Zeiss, Oberkochen, Germany) and analyzed using ImageJ (National Institutes of Health, Bethesda, MD).

## 2. Results

Overall, a likely pathogenic variant was found in 16 individuals in the cohort (16 of 52, 30.8%).

### A. Complete Gonadal Dysgenesis

**Figure 2.**
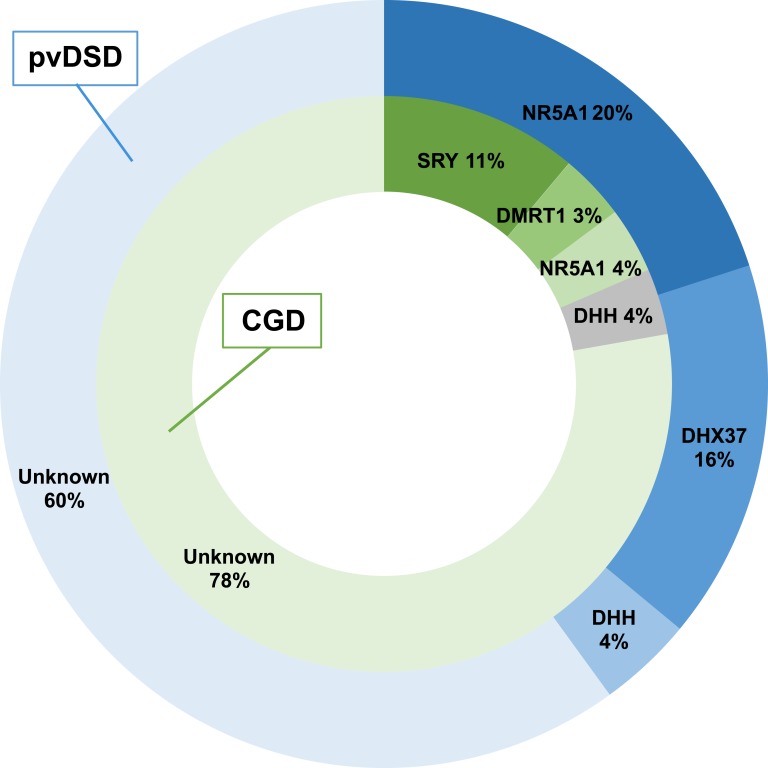
Overview of genetic diagnoses reached.

A molecular etiology was attained in 6 of 27 (22.2%) individuals with a clinical diagnosis of CGD (Figs. 1 and 2; Table 2)

**Table 2.** Overview of Clinical Features and Pathogenic Variants Identified in the Study Cohort

Working Diagnosis	Genital Phenotype	Müllerian Structures	Gene	Sequence Variation	Genotype	Inheritance	gnomAD Allele Frequency	SIFT	PolyPhen-2	Mutation Taster
CGD	Female	Present	SRY	p.R62P	Hemizygous	Sporadic	0	Damaging (0)	Probably damaging (1)	Disease causing
				c.185G>C						
CGD	Female	Present	SRY	p.N65D	Hemizygous	Sporadic	0	Damaging (0)	Probably damaging (0.998)	Disease causing
				c.193A>G						
CGD	Female	Present	SRY	p.L204fs*211 p.L204PLDKANG*	Hemizygous	Sporadic	0	N/A	N/A	N/A
CGD	Female	Present	DMRT1	p.R80S	Heterozygous	Sporadic	0	Damaging (0)	Probably damaging (0.997)	Disease causing
				c.240G>C						
CGD	Female	Present	NR5A1	p.A280E	Heterozygous	Sporadic	0	Damaging (0)	Probably damaging (1)	Disease causing
				c.839C>A						
CGD	Female	Present	DHH	P.F242L	Homozygous	Sporadic	0	Damaging (0.014)	Probably damaging (0.997)	Disease causing
				c.724T>C						
pvDSD	Partially virilized	Absent	NR5A1*^a^*	p.G22D	Heterozygous	Sporadic	0	Damaging (0)	Probably damaging (1)	Disease causing
				c.65G>A						
pvDSD	Partially virilized	Absent	NR5A1	p.R281C	Heterozygous	Sporadic	0	Damaging (0)	Probably damaging (1)	Disease causing
				c.841C>T						
pvDSD	Partially virilized	Absent	NR5A1	p.G328R c.982G>C	Heterozygous	Sporadic	0	Damaging (0)	Probably damaging (1)	Disease causing
pvDSD	Partially virilized	Absent	NR5A1	p.E367Sfs*15 c.1099delG	Heterozygous	Sporadic	0	N/A	N/A	N/A
pvDSD	Virilization at puberty	Absent	NR5A1	p.L420P	Heterozygous	Familial (SLD)	0	Damaging (0)	Probably damaging (1)	Disease causing
				c.1259T>C						
pvDSD	Partially virilized, further virilization at puberty	Absent	DHH	p.R245P	Compound heterozygous	Sporadic	0	Damaging (0.002)	Probably damaging (0.999)	Disease causing
				c.734G>C						
				p.A227V			2/30,956	Tolerated (0.101)	Possibly damaging (0.904)	Disease causing
				c.680C>T						
pvDSD	Partially virilized	Absent	DHX37*^b^*	p.R308Q	Heterozygous	Sporadic	1/30,936	Damaging (0)	Probably damaging (1)	Disease causing
				c.923G>A						
pvDSD	Partially virilized	Absent	DHX37*^b^*	p.R308Q	Heterozygous	Sporadic	1/30,936	Damaging (0)	Probably damaging (1)	Disease causing
				c.923G>A						
pvDSD	Partially virilized	Vaginal septum/uterine didelphys	DHX37*^b^*	p.R308Q	Heterozygous	Sporadic	1/30,936	Damaging (0)	Probably damaging (1)	Disease causing
				c.923G>A						
pvDSD	Partially virilized	Absent	DHX37	p.T477M	Heterozygous	Sporadic	0	Damaging (0)	Probably damaging (1)	Disease causing
				c.1430C>T						

Abbreviations: N/A, not applicable; SLD, sex-limited dominant.

^a^ Also heterozygous for a c.1305G>T, p.E435D variant that is predicted to be benign.

^b^ Previously published [40].

**Figure 3.**
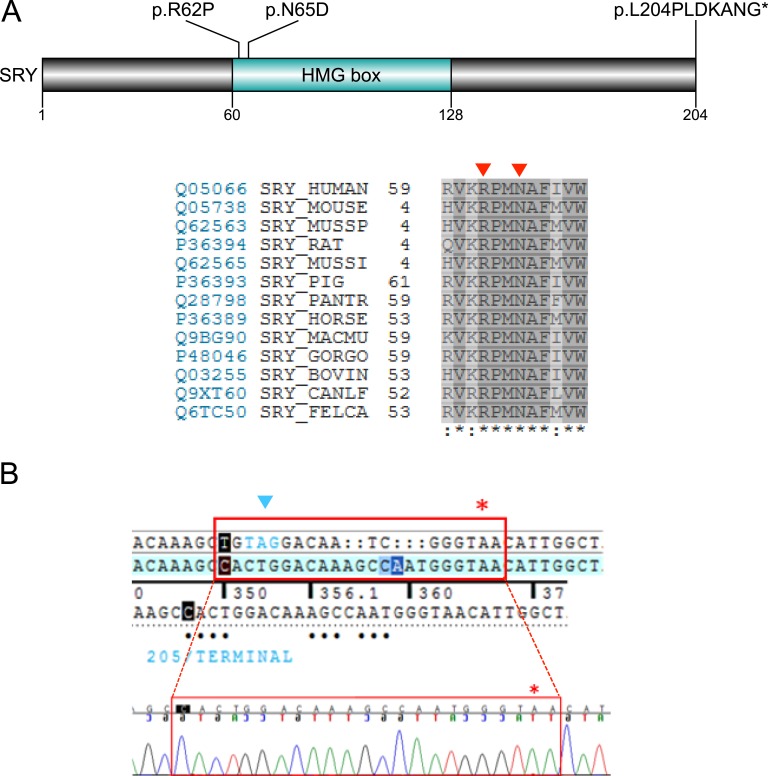
(A) Depiction of SRY demonstrating the mutations identified, with amino acid conservancy shown below. The position of variants is indicated by red arrowheads. (B) Aligned sequence and chromatogram showing the complex indel variant. The position of the typical stop codon at 205 is shown with a blue arrow. The red asterisk indicates where the frameshift ends. HMG, high-mobility group.

Three individuals had hemizygous pathogenic variants in SRY sex-determining region Y (SRY), with two changes affecting codons in the high-mobility group box (p.R62P, p.N65D) and one variant being a novel, complex insertion-deletion affecting the native stop codon of SRY (Table 2; Fig. 3A and 3B).

**Figure 4.**
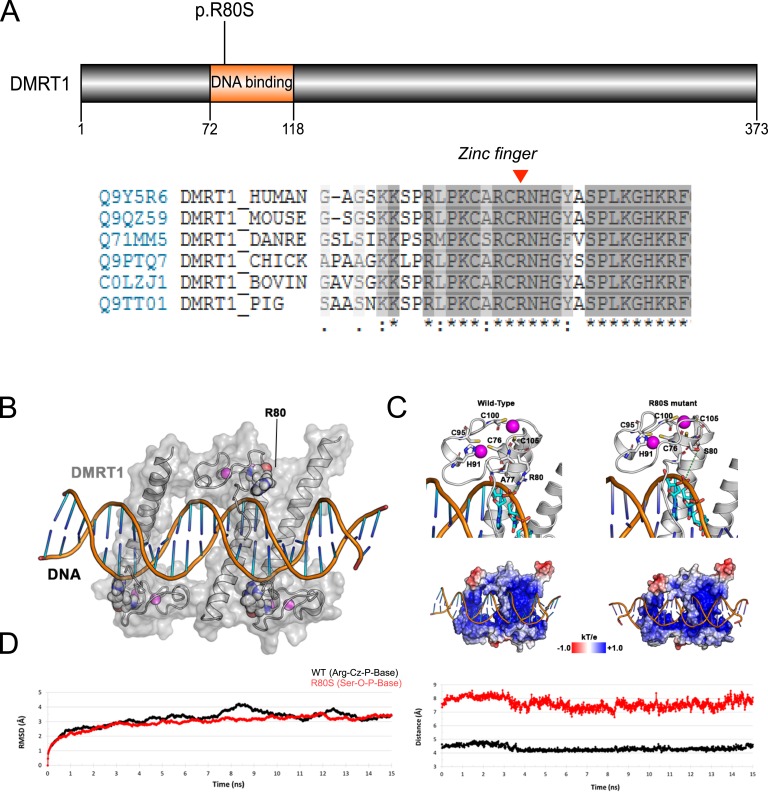
(A) Depiction of DMRT1 demonstrating the p.R80S mutation identified, with amino acid conservancy shown below. The position of p.R80 is indicated by a red arrowhead. (B) Schematic representation of the DMRT1–DNA complex (PDB ID 4YJ0). The DMRT1 protein is shown in white and the R80 residues are depicted as spheres; zinc atoms are shown in magenta. (C) Snapshot of the R80 or S80 vicinity (upper) and electrostatic potential of surface of the respective DMRT1-DNA complexes (lower). (D) C*α*-root-mean-squared (RMSD) deviation and distances between Arg or Ser 80 and the phosphate backbone of the DNA along the molecular dynamics simulation.

One individual with CGD was found to have a heterozygous p.R80S variant in the first zinc finger of DMRT1 (Table 2; Fig. 4A). This variant is predicted to be damaging and was not found in the gnomAD database (Table 2). To gain insight into the potential effect this variant has on DMRT1, detailed molecular dynamics simulation of both WT DMRT and the p.R80S mutant was performed. The R80 residue interfaces with DNA, binding through a hydrogen bond (H-bond) interaction with the phosphate backbone of thymidine 16 (Fig. 4B and 4C). The p.R80S mutant loses H-bond contacts with this phosphate backbone of DNA and displays longer distances compared with WT (7.63 ± 0.36 Å vs 4.34 ± 0.18 Å) during simulation time (Fig. 4C and 4D). The electrostatic potential of the surface does not change significantly (Fig. 4C).

**Figure 5.**
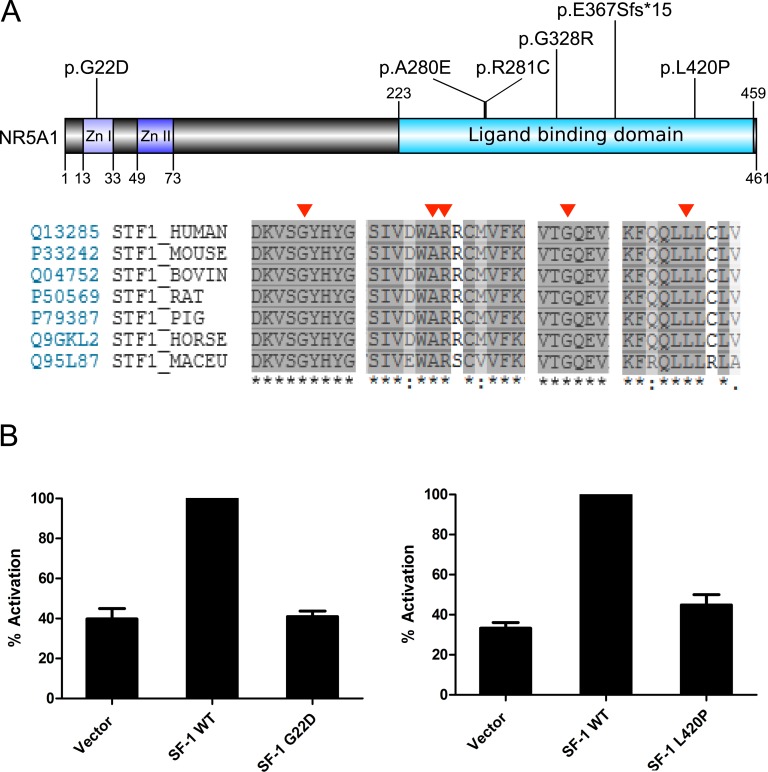
(A) Depiction of NR5A1 (SF-1) demonstrating the mutations identified, with amino acid conservancy shown below. The positions of variants are indicated by red arrowheads. (B) Transient transfection assays showing activation of a Cyp11a1 promoter by WT SF-1 and impaired transcriptional activity by the p.G22D and p.L420P mutants. Results are shown as a percentage of WT SF-1 activity (relative light units). Data are shown as mean ±SEM of three experiments performed in triplicate. Zn, Zinc finger.

One woman diagnosed with CGD was found to harbor a heterozygotic p.A280E variant in helix 3 of the ligand-binding domain (LBD) of SF-1 (NR5A1) (Table 2; Fig. 5A).

**Figure 6.**
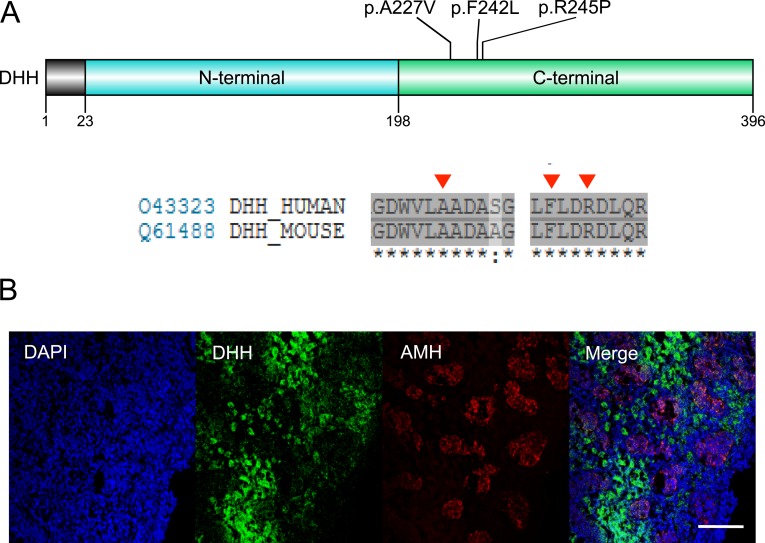
(A) Depiction of DHH demonstrating the mutations identified, with amino acid conservancy shown below. The positions of variants are indicated by red arrowheads. (B) Immunohistochemistry of human fetal testis at 9 wk postconception showing expression of DHH (green) predominantly in interstitial Leydig cells but also in Sertoli cells. Anti-Müllerian hormone (AMH; red) is expressed strongly in Sertoli cells of primitive seminiferous tubules. Nuclei are stained blue with 4′,6-diamidino-2-phenylindole (DAPI). No staining is seen in the peripheral capsular region at the bottom right of the image. Scale bar, 100 μm.

Another woman with primary amenorrhea and absent uterus was found to have a homozygous p.F242L variant in DHH (Table 2; Fig. 6A).

### B. pvDSD

A molecular diagnosis was attained in a greater proportion of individuals with a clinical phenotype of pvDSD (10 of 25; 40.0%) compared with CGD (Figs. 1 and 2; Table 2).

Five pathogenic variants, all in the heterozygous state, were found in *NR5A1* (p.G22D, p.R281C, p.G328R, p.E367Sfs*15, p.L420P) (Table 2; Fig. 5A). These include four missense changes and a frameshift change affecting codons in the LBD. Transient transfection studies confirmed that two of these NR5A1 missense variants (p.G22D and p.L420P) likely cause loss of function (Fig. 5B), whereas the other two variants affect codons previously shown to be disrupted in individuals with DSD.

One woman with partial virilization and absent Müllerian structures was found to have compound heterozygous (p.A227V/p.R245P) variants in DHH, affecting a fairly localized region within the C-terminal domain close to the p.F242 codon described above (Table 2; Fig. 6A). Using IHC, we showed strong expression of DHH in interstitial cells of the testis just after the establishment of androgen biosynthesis in the human fetus (9 weeks postconception), with weaker expression in the Sertoli cells of primitive seminiferous tubules (Fig. 6B).

**Figure 7.**
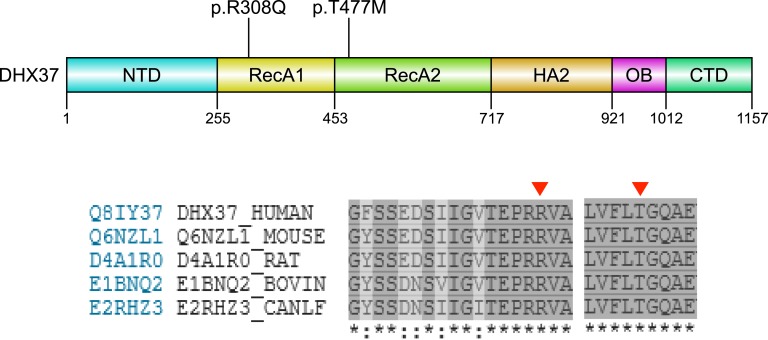
Depiction of DHX37 demonstrating the mutations identified, with amino acid conservancy shown below. The positions of variants are indicated by red arrowheads. CTD, C-terminal domain; HA2, helicase-associated 2 domain; NTD, N-terminal domain; OB, oligonucleotide/oligosaccharide-like domain; RecA1, ATP binding DEAH box helicase; RecA2, C-terminal helicase.

Finally, four individuals had pathogenic heterozygous variants in DEAH-box helicase 37 (DHX37). Three of these affect a p.R308Q hotspot, and these have recently been reported separately (Table 2; Fig. 7) [40]. One additional variant (p.T477M) was identified, which affects a highly conserved amino acid in the Rec-A2 motif IV RNA-binding region and is predicted to be damaging and disease causing.

### C. Candidate Genes for DSD

Analysis of candidate genes for DSD based on our experience of transcriptomic profiling did not reveal any clear pathogenic variants in those genes analyzed (Table 1). No likely pathogenic changes were found in the three sibling pairs studied.

## 3. Discussion

Reaching a specific diagnosis in 46,XY DSD can be challenging, as there are many different potential causes and, historically, these may have been grouped under umbrella terms such as Swyer syndrome (CGD) or pvDSD [41].

Although many of these patients have classic conditions such as 17*β*-HSD, 5ARD, and AIS, individuals may not have been fully investigated initially, and making a diagnosis can be difficult when the testes have been removed [5, 6]. Reaching a specific diagnosis can sometimes have benefits for understanding the natural history of a condition, identifying associated features, defining likely inheritance and chances of other family members being affected, and in the long term for understanding tumor risk [8]. Therefore, a molecular approach to diagnosis is becoming increasingly important in both research and clinical settings [4, 8, 20].

In this study, we report our analysis of a large cohort of 46,XY women with DSD from a single center. Using targeted genetic analysis and biochemical profiling (*e.g.*, urine steroid profiling by gas chromatography–tandem mass spectrometry), those with 17*β*-HSD, 5ARD and AIS had been previously screened out, leaving a cohort of >50 women who were recruited with a working diagnosis of CGD or pvDSD [5, 6]. This is a typical scenario in clinical practice and will be increasingly common as adult DSD services, and teams are established to provide better long-term follow-up and support in a multidisciplinary setting [42–44].

Using an NGS targeted sequencing approach of known and candidate genes, we were able to reach a likely molecular diagnosis in 30.8% of the cohort as a whole, with a higher proportion of genetic diagnoses being reached in the pvDSD group (40.0%) compared with the CGD group (22.2%). This is similar to the other limited data available, but importantly note, that many studies to date have included individuals with conditions such as 17*β*-HSD, 5ARD, and AIS, and they have been less stringent than the current study that focuses on a cohort of adults with undiagnosed DSD.

Within our study cohort we found likely pathogenic variants in five different genes (*SRY, DMRT1, NR5A1, DHH, DHX37*), with some overlap in presentation between the two broad diagnostic groups. Our findings also provide insight into molecular aspects of these conditions.

As expected, hemizygous variants in SRY were found in three women with CGD who had presented with absent puberty and primary amenorrhea in adolescence. The prevalence of 11.5% of CGD is consistent with previous data and, as expected, the two missense variants (p.R62P, p.N65D) affect amino acids in the high-mobility group box of SRY, potentially in the region of a nuclear localization signal or calmodulin-binding motif [45, 46]. Of interest, a third individual had a complex insertion-deletion event that removes the native stop codon of SRY and is predicted to result in the translation of a protein with an additional seven amino acids at the C-terminal end (p.L204fs*211 p.L204PLDKANG*). This alteration was not defined clearly on NGS sequencing, and a Sanger sequencing approach was needed to clarify the exact changes (Fig. 3B).

One individual with CGD was found to harbor a heterozygous p.R80S variant in the first zinc finger of DMRT1. DMRT1 has been known for several years to play a key role in sex determination in different species, but its role in human sex development beyond the 10q23 deletion syndrome is less clear [47]. More recently, a variant in DMRT1 (p.R111G) has been reported in association with testicular dysgenesis that affects the DMRT1 recognition helix [28]. Using a similar approach, it is likely that the p.R80S variant affects interactions between DMRT1 and the minor groove of DNA (Fig. 4B–4D). As well as being located in a key domain, this variant is highly conserved among species, is predicted to be damaging, and is not present in population databases. Using molecular modeling and simulation, we have shown that substitution of the larger, polar arginine residue with the smaller serine disrupts an H-bond between DMRT1 and DNA. It is expected that an additive effect on the loss of affinity that will depend on the stoichiometry of the DMRT1–DNA complex occurs, resulting in the final phenotypic effect. Of note, Wang *et al.* [14] recently reported a p.Y84C variant in DMRT1 associated with DSD, but concluded that the significance was unknown.

Six individuals in our cohort were found to have heterozygous missense (n = 5) or frameshift (n = 1) changes in SF-1 (NR5A1). SF-1/NR5A1 is a nuclear receptor transcription factor that was originally shown to be important in adrenal and gonad development [48]. However, it has emerged in the past 15 years that variants in SF-1/NR5A1 are one of the most common causes of 46,XY DSD, with phenotypes ranging from a complete gonadal dysgenesis scenario through various degrees of virilization and hypospadias to male factor infertility [10, 11, 22, 49]. Most changes occur *de novo* or in a sex-limited dominant pattern, although primary ovarian insufficiency can also occur [50].

The prevalence of SF-1/NR5A1 variants in our cohort (6 of 52, 11.5%) is similar to other studies, with an enrichment seen in the pvDSD subgroup (19.2%) [22]. One of the probands had a family history consistent with sex-limited dominant inheritance. One of the variants discovered (p.G22D) affects the DNA-binding domain of SF-1/NR5A1 and was shown to impair SF-1 function, as did the p.L420P variant in the LBD (Fig. 5B). The other missense variants identified (p.A280E, p.R281C, p.G328R) have either been reported previously or affect previously disrupted codons, and all are predicted to be damaging [10, 51–53]. Although it is not clear whether SF-1 has a true biological ligand, crystallization has shown that codons 280/281 form a key region of helix 3 that interacts with corepressors such as NR0B1/NR0B2 (DAX-1, SHP) [54]. Our findings confirm that variants in SF-1/NR5A1 are still the most prevalent cause of 46,XY DSD currently known.

Another key gene emerging as a relatively prevalent cause of DSDs is *DHH* [55–61]. We identified DHH changes in two individuals with a recessive form of DSD who had homozygous (p.F242L) or compound heterozygous (p.A227V/p.R245P) variants, and with differing phenotypes. Disruption of DHH is sometimes associated with minifascicular neuropathy and was thought potentially to affect testicular interstitial/Leydig cell development in mice and humans, as well as Leydig–Sertoli cell interactions [56, 59, 62]. Furthermore, we have shown by IHC that DHH is expressed predominantly in developing Leydig cells in the human fetal testis at a critical early stage of development just after the onset of steroidogenesis (9 weeks postconception) (Fig. 6B) [21]. However, DHH may also play a role in Sertoli cells and Leydig–Sertoli cell interactions, as areas of gonadal dysgenesis or streak gonads have been reported and Dhh is expressed in mouse Sertoli cells in single-cell RNA sequencing, and DHH expression was seen in Sertoli cells, albeit at a lower level (Fig. 6B) [56, 59, 63, 64]. Consistent with this variability, one woman had a CGD phenotype with a functional uterus and streak gonads, whereas the other had partially virilized genitalia and no Müllerian structures. The variants identified are located within a fairly localized region of DHH, but relatively little is known about the structural or biological effects of these changes.

Finally, four individuals (7.7% overall; 15.4% of pvDSD) in our cohort had heterozygous disruptive variants in DHX37. DHX37 is an RNA helicase and predicted ribosomal RNA-binding protein, but its exact biological function in testis development is still unclear. Variants in DHX37 are emerging as a relatively prevalent cause of a range of DSD phenotypes, including vanishing testis syndrome [40, 65]. Three individuals in our cohort were found to have a recurrent p.R308Q variant in the RecA1 motif, and they have been included in a recent series describing the role of DHX37 in 46,XY DSD [40]. The p.R308Q variant is often *de novo* and has been found in diverse ancestral backgrounds. One additional person in our cohort with mild virilization and an absent uterus was found to have a p.T477M variant located within the RecA2 motif IV involved in RNA binding (Fig. 7). This variant occurs in a highly conserved codon/region and is a predicted disruptive event. Future studies are needed to elucidate the role of DHX37 in sex development, but the association of variants in this gene with 46,XY DSD is becoming well established.

Although we did not expect to discover variants involved in proximal steroidogenic blocks with adrenal or biochemical phenotypes (*e.g.*, STAR, CYP11A1, HSD3B2, CYP17A1, POR, CYB5A) or associated with specific features (*e.g.*, SOX9, campomelic dysplasia; GATA4/ZFPM2, cardiac; WT1, renal), we did not find likely pathogenic variants in other DSD-associated genes such as MAP3K1, SOX8, ESR2, or ZNRF3. Furthermore, no clear pathogenic variants were found in candidate genes based on our transcriptomic studies of genes expressed in early human testis development [21]. In these studies, we used a modeling approach to identify genes that were either upregulated in a time-series data set with similar patterns to SOX9 (*e.g.*, CITED1), or that were differentially expressed fetal testis genes or potential novel components of steroidogenesis. Although it might be expected to find variants in some of these candidate factors, in fact no clear causative defects were identified.

This study has certain limitations. First, a panel-based approach means that this study is more effective at identifying the relative prevalence of changes in known genes in this cohort. Whole-exome and whole-genome sequencing approaches are better for gene discovery. As costs reduce, analysis becomes more robust and more accurate counseling can be provided about the risk of incidental findings, these technologies are becoming the first-line strategy in many situations, especially where multiple family members are affected. It remains possible that nongenomic events such as methylation defects or complex gene–environment interactions may cause some forms of DSD, especially complete testicular dysgenesis where the diagnostic rate is still relatively low. Second, functional assays are not well established for several of these factors, although we think that the genetic evidence for causality presented here is strong. Finally, studying a predominantly adult cohort means that access to historic data or family members is limited. However, it is increasingly being recognized that adult DSD services need to be established in parallel with multidisciplinary pediatric services, so this work provides useful insight into the range of diagnoses that might be made.

## 4. Conclusion

NGS approaches are improving the diagnostic yield in individuals with complete and partial forms of gonadal dysgenesis, or in those who have been labeled as having “partial androgen insensitivity” previously. Reaching a specific genetic diagnosis can inform genetic counseling, particularly for families with ongoing consanguinity, and can help to identify associated comorbiditites. Lastly, as we gain more information on the life course of adults with DSD, so we can seek correlations between genotype and phenotype especially with regard to late onset features such as osteoporosis.

## Appendix: Web Resources

Mutation Taster: http://www.mutationtaster.org/

PolyPhen-2: http://genetics.bwh.harvard.edu/pph2/

Sorting Intolerant From Tolerant (SIFT): https://sift.bii.a-star.edu.sg

Genome Aggregation Database: http://gnomad.broadinstitute.org/

Ingenuity variant analysis: http://www.ingenuity.com/

UniProt: https://www.uniprot.org

## Additional Information

***Disclosure Summary:*** The authors have nothing to disclose.

***Data Availability:*** The datasets generated during and/or analyzed during the current study are not publicly available but are available from the corresponding author on reasonable request.

## Abbreviations:

5ARD: 5*α*-reductase deficiency
17*β*-HSD: 17*β*-hydroxysteroid dehydrogenase deficiency type 3
AIS: androgen insensitivity syndrome
CGD: complete gonadal dysgenesis
DHH: desert hedgehog
DHX37: DEAH-box helicase 37
DMRT1: doublesex and mab-3–related transcription factor 1
DSD: difference (disorder) of sex development
gnomAD: Genome Aggregation Database
H-bond: hydrogen bond
IHC: immunohistochemistry
LBD: ligand-binding domain
NGS: next-generation sequencing
PAIS: partial androgen insensitivity syndrome
PGD: partial gonadal dysgenesis
pvDSD: partially virilized 46,XY disorder of sex development
SRY: sex-determining region Y
TBS: Tris-buffered saline
WT: wild-type

## References

[1] Sinclair AH, Berta P, Palmer MS, Hawkins JR, Griffiths BL, Smith MJ, Foster JW, Frischauf AM, Lovell-Badge R, Goodfellow PN A gene from the human sex-determining region encodes a protein with homology to a conserved DNA-binding motif. Nature. 1990;346(6281):240–244. 1695712 10.1038/346240a0

[2] Berta P, Hawkins JR, Sinclair AH, Taylor A, Griffiths BL, Goodfellow PN, Fellous M Genetic evidence equating SRY and the testis-determining factor. Nature. 1990;348(6300):448–450. 2247149 10.1038/348448A0

[3] Koopman P, Münsterberg A, Capel B, Vivian N, Lovell-Badge R Expression of a candidate sex-determining gene during mouse testis differentiation. Nature. 1990;348(6300):450–452. 2247150 10.1038/348450a0

[4] Audí L, Ahmed SF, Krone N, Cools M, McElreavey K, Holterhus PM, Greenfield A, Bashamboo A, Hiort O, Wudy SA, McGowan R; The EU COST Action. Genetics in endocrinology: approaches to molecular genetic diagnosis in the management of differences/disorders of sex development (DSD): position paper of EU COST Action BM 1303 “DSDnet”. Eur J Endocrinol. 2018;179(4):R197–R206.30299888

[5] Phelan N, Williams EL, Cardamone S, Lee M, Creighton SM, Rumsby G, Conway GS Screening for mutations in 17β-hydroxysteroid dehydrogenase and androgen receptor in women presenting with partially virilised 46,XY disorders of sex development. Eur J Endocrinol. 2015;172(6):745–751. 25740850 10.1530/EJE-14-0994

[6] Berra M, Williams EL, Muroni B, Creighton SM, Honour JW, Rumsby G, Conway GS Recognition of 5α-reductase-2 deficiency in an adult female 46XY DSD clinic. Eur J Endocrinol. 2011;164(6):1019–1025. 21402750 10.1530/EJE-10-0930

[7] Hughes IA, Houk C, Ahmed SF, Lee PA; LWPES Consensus Group; ESPE Consensus Group. Consensus statement on management of intersex disorders. Arch Dis Child. 2006;91(7):554–563. 16624884 10.1136/adc.2006.098319 PMC2082839

[8] Achermann JC, Domenice S, Bachega TA, Nishi MY, Mendonca BB Disorders of sex development: effect of molecular diagnostics. Nat Rev Endocrinol. 2015;11(8):478–488. 25942653 10.1038/nrendo.2015.69

[9] Ahmed SF, Achermann JC, Arlt W, Balen A, Conway G, Edwards Z, Elford S, Hughes IA, Izatt L, Krone N, Miles H, O’Toole S, Perry L, Sanders C, Simmonds M, Watt A, Willis D Society for Endocrinology UK guidance on the initial evaluation of an infant or an adolescent with a suspected disorder of sex development (revised 2015). Clin Endocrinol (Oxf). 2016;84(5):771–788. 26270788 10.1111/cen.12857 PMC4855619

[10] Suntharalingham JP, Buonocore F, Duncan AJ, Achermann JC DAX-1 (NR0B1) and steroidogenic factor-1 (SF-1, NR5A1) in human disease. Best Pract Res Clin Endocrinol Metab. 2015;29(4):607–619. 26303087 10.1016/j.beem.2015.07.004 PMC5159745

[11] Domenice S, Machado AZ, Ferreira FM, Ferraz-de-Souza B, Lerario AM, Lin L, Nishi MY, Gomes NL, da Silva TE, Silva RB, Correa RV, Montenegro LR, Narciso A, Costa EM, Achermann JC, Mendonca BB Wide spectrum of NR5A1-related phenotypes in 46,XY and 46,XX individuals. Birth Defects Res C Embryo Today. 2016;108(4):309–320. 28033660 10.1002/bdrc.21145 PMC5347970

[12] De Franco E, Flanagan SE, Houghton JA, Lango Allen H, Mackay DJ, Temple IK, Ellard S, Hattersley AT The effect of early, comprehensive genomic testing on clinical care in neonatal diabetes: an international cohort study. Lancet. 2015;386(9997):957–963. 26231457 10.1016/S0140-6736(15)60098-8 PMC4772451

[13] Guran T, Buonocore F, Saka N, Ozbek MN, Aycan Z, Bereket A, Bas F, Darcan S, Bideci A, Guven A, Demir K, Akinci A, Buyukinan M, Aydin BK, Turan S, Agladioglu SY, Atay Z, Abali ZY, Tarim O, Catli G, Yuksel B, Akcay T, Yildiz M, Ozen S, Doger E, Demirbilek H, Ucar A, Isik E, Ozhan B, Bolu S, Ozgen IT, Suntharalingham JP, Achermann JC Rare causes of primary adrenal insufficiency: genetic and clinical characterization of a large nationwide cohort. J Clin Endocrinol Metab. 2016;101(1):284–292. 26523528 10.1210/jc.2015-3250 PMC4701852

[14] Wang H, Zhang L, Wang N, Zhu H, Han B, Sun F, Yao H, Zhang Q, Zhu W, Cheng T, Cheng K, Liu Y, Zhao S, Song H, Qiao J Next-generation sequencing reveals genetic landscape in 46,XY disorders of sexual development patients with variable phenotypes. Hum Genet. 2018;137(3):265–277. 29582157 10.1007/s00439-018-1879-y

[15] Eggers S, Sadedin S, van den Bergen JA, Robevska G, Ohnesorg T, Hewitt J, Lambeth L, Bouty A, Knarston IM, Tan TY, Cameron F, Werther G, Hutson J, O’Connell M, Grover SR, Heloury Y, Zacharin M, Bergman P, Kimber C, Brown J, Webb N, Hunter MF, Srinivasan S, Titmuss A, Verge CF, Mowat D, Smith G, Smith J, Ewans L, Shalhoub C, Crock P, Cowell C, Leong GM, Ono M, Lafferty AR, Huynh T, Visser U, Choong CS, McKenzie F, Pachter N, Thompson EM, Couper J, Baxendale A, Gecz J, Wheeler BJ, Jefferies C, MacKenzie K, Hofman P, Carter P, King RI, Krausz C, van Ravenswaaij-Arts CM, Looijenga L, Drop S, Riedl S, Cools M, Dawson A, Juniarto AZ, Khadilkar V, Khadilkar A, Bhatia V, Dũng VC, Atta I, Raza J, Thi Diem Chi N, Hao TK, Harley V, Koopman P, Warne G, Faradz S, Oshlack A, Ayers KL, Sinclair AH Disorders of sex development: insights from targeted gene sequencing of a large international patient cohort. Genome Biol. 2016;17(1):243. 27899157 10.1186/s13059-016-1105-y PMC5126855

[16] Hughes LA, McKay Bounford K, Webb E, Dasani P, Clokie S, Chandran H, McCarthy L, Mohamed Z, Kirk JM, Krone N, Allen S, Cole T Next generation sequencing (NGS) to improve the diagnosis and management of patients with disorders of sex development (DSD). Endocr Connect. 2019;8(2):100–110. 10.1530/EC-18-0376 PMC6373624 30668521

[17] Kim JH, Kang E, Heo SH, Kim GH, Jang JH, Cho EH, Lee BH, Yoo HW, Choi JH Diagnostic yield of targeted gene panel sequencing to identify the genetic etiology of disorders of sex development. Mol Cell Endocrinol. 2017;444:19–25. 28130116 10.1016/j.mce.2017.01.037

[18] Dong Y, Yi Y, Yao H, Yang Z, Hu H, Liu J, Gao C, Zhang M, Zhou L, Asan, Yi X, Liang Z Targeted next-generation sequencing identification of mutations in patients with disorders of sex development. BMC Med Genet. 2016;17(1):23. 26980296 10.1186/s12881-016-0286-2 PMC4791760

[19] Baxter RM, Arboleda VA, Lee H, Barseghyan H, Adam MP, Fechner PY, Bargman R, Keegan C, Travers S, Schelley S, Hudgins L, Mathew RP, Stalker HJ, Zori R, Gordon OK, Ramos-Platt L, Pawlikowska-Haddal A, Eskin A, Nelson SF, Délot E, Vilain E Exome sequencing for the diagnosis of 46,XY disorders of sex development. J Clin Endocrinol Metab. 2015;100(2):E333–E344. 25383892 10.1210/jc.2014-2605 PMC4318895

[20] Délot EC, Papp JC, Sandberg DE, Vilain E; DSD-TRN Genetics Workgroup. Genetics of disorders of sex development: the DSD-TRN experience. Endocrinol Metab Clin North Am. 2017;46(2):519–537. 28476235 10.1016/j.ecl.2017.01.015 PMC5714504

[21] Del Valle I, Buonocore F, Duncan AJ, Lin L, Barenco M, Parnaik R, Shah S, Hubank M, Gerrelli D, Achermann JC A genomic atlas of human adrenal and gonad development. Wellcome Open Res. 2017;2:25. 28459107 10.12688/wellcomeopenres.11253.2 PMC5407452

[22] Lin L, Philibert P, Ferraz-de-Souza B, Kelberman D, Homfray T, Albanese A, Molini V, Sebire NJ, Einaudi S, Conway GS, Hughes IA, Jameson JL, Sultan C, Dattani MT, Achermann JC Heterozygous missense mutations in steroidogenic factor 1 (SF1/Ad4BP, *NR5A1*) are associated with 46,XY disorders of sex development with normal adrenal function. J Clin Endocrinol Metab. 2007;92(3):991–999. 17200175 10.1210/jc.2006-1672 PMC1872053

[23] Robinson JT, Thorvaldsdóttir H, Wenger AM, Zehir A, Mesirov JP Variant review with the integrative genomics viewer. Cancer Res. 2017;77(21):e31–e34. 29092934 10.1158/0008-5472.CAN-17-0337 PMC5678989

[24] Karczewski KJ, Francioli LC, Tiao G, Cummings BB, Alföldi J, Wang Q, Collins RL, Laricchia KM, Ganna A, Birnbaum DP, Gauthier LD, Brand H, Solomonson M, Watts NA, Rhodes D, Singer-Berk M, Seaby EG, Kosmicki JA, Walters RK, Tashman K, Farjoun Y, Banks E, Poterba T, Wang A, Seed C, Whiffin N, Chong JX, Samocha KE, Pierce-Hoffman E, Zappala Z, O’Donnell-Luria AH, Minikel EV, Weisburd B, Lek M, Ware JS, Vittal C, Armean IM, Bergelson L, Cibulskis K, Connolly KM, Covarrubias M, Donnelly S, Ferriera S, Gabriel S, Gentry J, Gupta N, Jeandet T, Kaplan D, Llanwarne C, Munshi R, Novod S, Petrillo N, Roazen D, Ruano-Rubio V, Saltzman A, Schleicher M, Soto J, Tibbetts K, Tolonen C, Wade G, Talkowski ME, Consortium TGAD, Neale BM, Daly MJ, MacArthur DG; The Genome Aggregation Database Consortium. Variation across 141,456 human exomes and genomes reveals the spectrum of loss-of-function intolerance across human protein-coding genes. bioRxiv. 2019 Deposited 1 April 2019. doi: https://doi.org/10.1101/531210.

[25] Ren J, Wen L, Gao X, Jin C, Xue Y, Yao X DOG 1.0: illustrator of protein domain structures. Cell Res. 2009;19(2):271–273. 19153597 10.1038/cr.2009.6

[26] Webb B, Sali A Protein structure modeling with MODELLER. Methods Mol Biol. 2014;1137:1–15. 24573470 10.1007/978-1-4939-0366-5_1

[27] UniProt Consortium. UniProt: a hub for protein information. Nucleic Acids Res. 2015;43(Database issue):D204–D212. 25348405 10.1093/nar/gku989 PMC4384041

[28] Murphy MW, Lee JK, Rojo S, Gearhart MD, Kurahashi K, Banerjee S, Loeuille GA, Bashamboo A, McElreavey K, Zarkower D, Aihara H, Bardwell VJ An ancient protein–DNA interaction underlying metazoan sex determination. Nat Struct Mol Biol. 2015;22(6):442–451. 26005864 10.1038/nsmb.3032 PMC4476070

[29] Humphrey W, Dalke A, Schulten K VMD: visual molecular dynamics. J Mol Graph. 1996;14(1):33–38, 27–28. 8744570 10.1016/0263-7855(96)00018-5

[30] Jorgensen WL, Chandrasekhar J, Madura JD, Impey RW, Klein ML Comparison of simple potential functions for simulating liquid water. J Chem Phys. 1983;79(2):926–935.

[31] Essmann U, Perera L, Berkowitz ML, Darden T, Lee H, Pedersen LG A smooth particle mesh Ewald method. J Chem Phys. 1995;103(19):8577–8593.

[32] Phillips JC, Braun R, Wang W, Gumbart J, Tajkhorshid E, Villa E, Chipot C, Skeel RD, Kalé L, Schulten K Scalable molecular dynamics with NAMD. J Comput Chem. 2005;26(16):1781–1802. 16222654 10.1002/jcc.20289 PMC2486339

[33] Best RB, Zhu X, Shim J, Lopes PEM, Mittal J, Feig M, Mackerell AD Jr Optimization of the additive CHARMM all-atom protein force field targeting improved sampling of the backbone φ, ψ and side-chain χ_1_ and χ_2_ dihedral angles. J Chem Theory Comput. 2012;8(9):3257–3273. 23341755 10.1021/ct300400x PMC3549273

[34] Klauda JB, Venable RM, Freites JA, O’Connor JW, Tobias DJ, Mondragon-Ramirez C, Vorobyov I, MacKerell AD Jr, Pastor RW Update of the CHARMM all-atom additive force field for lipids: validation on six lipid types. J Phys Chem B. 2010;114(23):7830–7843. 20496934 10.1021/jp101759q PMC2922408

[35] Feller SE, Zhang Y, Pastor RW, Brooks BR Constant pressure molecular dynamics simulation: the Langevin piston method. J Chem Phys. 1995;103(11):4613–4621.

[36] RRID:AB_2091715, https://scicrunch.org/resolver/AB_2091715.

[37] RRID:AB_10711946, https://scicrunch.org/resolver/AB_10711946.

[38] RRID:AB_2534069, https://scicrunch.org/resolver/AB_2534069.

[39] RRID:AB_141761, https://scicrunch.org/resolver/AB_141761.

[40] McElreavey K, Jorgensen A, Eozenou C, Merel T, Bignon-Topalovic J, Tan DS, Houzelstein D, Buonocore F, Warr N, Kay RG, Peycelon M, Siffroi JP, Mazen I, Achermann JC, Shcherbak Y, Leger J, Sallai A, Carel JC, Martinerie L, Le Ru R, Conway GS, Mignot B, Van Maldergem L, Bertalan R, Globa E, Brauner R, Jauch R, Nef S, Greenfield A, Bashamboo A Pathogenic variants in the DEAH-box RNA helicase DHX37 are a frequent cause of 46,XY gonadal dysgenesis and 46,XY testicular regression syndrome [published online ahead of print 24 July 2019]. Genet Med. 2019. doi: 10.1038/s41436-019-0606-y. 10.1038/s41436-019-0606-y PMC6944638 31337883

[41] Minto CL, Crouch NS, Conway GS, Creighton SM XY females: revisiting the diagnosis. BJOG. 2005;112(10):1407–1410. 16167945 10.1111/j.1471-0528.2005.00664.x

[42] Cools M, Nordenström A, Robeva R, Hall J, Westerveld P, Flück C, Köhler B, Berra M, Springer A, Schweizer K, Pasterski V; COST Action BM1303 working group 1. Caring for individuals with a difference of sex development (DSD): a consensus statement. Nat Rev Endocrinol. 2018;14(7):415–429. 29769693 10.1038/s41574-018-0010-8 PMC7136158

[43] Falhammar H, Claahsen-van der Grinten H, Reisch N, Slowikowska-Hilczer J, Nordenström A, Roehle R, Bouvattier C, Kreukels BPC, Köhler B; dsd-LIFE group. Health status in 1040 adults with disorders of sex development (DSD): a European multicenter study. Endocr Connect. 2018;7(3):466–478. 29490934 10.1530/EC-18-0031 PMC5861372

[44] Rapp M, Mueller-Godeffroy E, Lee P, Roehle R, Kreukels BPC, Köhler B, Nordenström A, Bouvattier C, Thyen U; dsd-LIFE group. Multicentre cross-sectional clinical evaluation study about quality of life in adults with disorders/differences of sex development (DSD) compared to country specific reference populations (dsd-LIFE). Health Qual Life Outcomes. 2018;16(1):54. 29615040 10.1186/s12955-018-0881-3 PMC5883311

[45] Sim H, Argentaro A, Harley VR Boys, girls and shuttling of SRY and SOX9. Trends Endocrinol Metab. 2008;19(6):213–222. 18585925 10.1016/j.tem.2008.04.002

[46] Sim H, Argentaro A, Czech DP, Bagheri-Fam S, Sinclair AH, Koopman P, Boizet-Bonhoure B, Poulat F, Harley VR Inhibition of SRY-calmodulin complex formation induces ectopic expression of ovarian cell markers in developing XY gonads. Endocrinology. 2011;152(7):2883–2893. 21558314 10.1210/en.2010-1475

[47] Matson CK, Zarkower D Sex and the singular DM domain: insights into sexual regulation, evolution and plasticity. Nat Rev Genet. 2012;13(3):163–174. 22310892 10.1038/nrg3161 PMC3595575

[48] Achermann JC, Ito M, Ito M, Hindmarsh PC, Jameson JL A mutation in the gene encoding steroidogenic factor-1 causes XY sex reversal and adrenal failure in humans. Nat Genet. 1999;22(2):125–126. 10369247 10.1038/9629

[49] McElreavey K, Achermann JC Steroidogenic factor-1 (SF-1, NR5A1) and 46,XX ovotesticular disorders of sex development: one factor, many phenotypes. Horm Res Paediatr. 2017;87(3):189–190. 27978531 10.1159/000454806 PMC5569707

[50] Lourenço D, Brauner R, Lin L, De Perdigo A, Weryha G, Muresan M, Boudjenah R, Guerra-Junior G, Maciel-Guerra AT, Achermann JC, McElreavey K, Bashamboo A Mutations in *NR5A1* associated with ovarian insufficiency. N Engl J Med. 2009;360(12):1200–1210. 19246354 10.1056/NEJMoa0806228 PMC2778147

[51] Philibert P, Polak M, Colmenares A, Lortat-Jacob S, Audran F, Poulat F, Sultan C Predominant Sertoli cell deficiency in a 46,XY disorders of sex development patient with a new NR5A1/SF-1 mutation transmitted by his unaffected father. Fertil Steril. 2011;95(5):1788.e5–1788.e9. 10.1016/j.fertnstert.2010.11.035 21163476

[52] Tantawy S, Lin L, Akkurt I, Borck G, Klingmüller D, Hauffa BP, Krude H, Biebermann H, Achermann JC, Köhler B Testosterone production during puberty in two 46,XY patients with disorders of sex development and novel NR5A1 (SF-1) mutations. Eur J Endocrinol. 2012;167(1):125–130. 22474171 10.1530/EJE-11-0944 PMC3381348

[53] Rocca MS, Ortolano R, Menabò S, Baronio F, Cassio A, Russo G, Balsamo A, Ferlin A, Baldazzi L Mutational and functional studies on *NR5A1* gene in 46,XY disorders of sex development: identification of six novel loss of function mutations. Fertil Steril. 2018;109(6):1105–1113. 29935645 10.1016/j.fertnstert.2018.02.123

[54] Krylova IN, Sablin EP, Moore J, Xu RX, Waitt GM, MacKay JA, Juzumiene D, Bynum JM, Madauss K, Montana V, Lebedeva L, Suzawa M, Williams JD, Williams SP, Guy RK, Thornton JW, Fletterick RJ, Willson TM, Ingraham HA Structural analyses reveal phosphatidyl inositols as ligands for the NR5 orphan receptors SF-1 and LRH-1. Cell. 2005;120(3):343–355. 15707893 10.1016/j.cell.2005.01.024

[55] Umehara F, Tate G, Itoh K, Yamaguchi N, Douchi T, Mitsuya T, Osame M A novel mutation of desert hedgehog in a patient with 46,XY partial gonadal dysgenesis accompanied by minifascicular neuropathy. Am J Hum Genet. 2000;67(5):1302–1305. 11017805 10.1016/s0002-9297(07)62958-9 PMC1288570

[56] Werner R, Merz H, Birnbaum W, Marshall L, Schröder T, Reiz B, Kavran JM, Bäumer T, Capetian P, Hiort O 46,XY gonadal dysgenesis due to a homozygous mutation in desert hedgehog (*DHH*) identified by exome sequencing. J Clin Endocrinol Metab. 2015;100(7):E1022–E1029. 25927242 10.1210/jc.2015-1314 PMC4490300

[57] Paris F, Flatters D, Caburet S, Legois B, Servant N, Lefebvre H, Sultan C, Veitia RA A novel variant of *DHH* in a familial case of 46,XY disorder of sex development: Insights from molecular dynamics simulations. Clin Endocrinol (Oxf). 2017;87(5):539–544. 28708305 10.1111/cen.13420

[58] Baldinotti F, Cavallaro T, Dati E, Baroncelli GI, Bertini V, Valetto A, Massart F, Fabrizi GM, Zanette G, Peroni D, Bertelloni S Novel familial variant of the desert hedgehog gene: clinical findings in two Sisters with 46,XY gonadal dysgenesis or 46,XX karyotype and literature review. Horm Res Paediatr. 2018;89(3):141–149. 29471294 10.1159/000485507

[59] Rothacker KM, Ayers KL, Tang D, Joshi K, van den Bergen JA, Robevska G, Samnakay N, Nagarajan L, Francis K, Sinclair AH, Choong CS A novel, homozygous mutation in *desert hedgehog* (*DHH*) in a 46,XY patient with dysgenetic testes presenting with primary amenorrhoea: a case report. Int J Pediatr Endocrinol. 2018;2018(1):2. 29507583 10.1186/s13633-018-0056-3 PMC5834851

[60] Tajouri A, Kharrat M, Hizem S, Zaghdoudi H, M’rad R, Simic-Schleicher G, Kaiser FJ, Hiort O, Werner R In vitro functional characterization of the novel *DHH* mutations p.(Asn337Lysfs*24) and p.(Glu212Lys) associated with gonadal dysgenesis. Hum Mutat. 2018;39(12):2097–2109. 30298535 10.1002/humu.23664

[61] Ayers K, van den Bergen J, Robevska G, Listyasari N, Raza J, Atta I, Riedl S, Rothacker K, Choong C, Faradz SMH, Sinclair A Functional analysis of novel desert hedgehog gene variants improves the clinical interpretation of genomic data and provides a more accurate diagnosis for patients with 46,XY differences of sex development. J Med Genet. 2019;56(7):434–443. 31018998 10.1136/jmedgenet-2018-105893 PMC6591740

[62] Yao HHC, Whoriskey W, Capel B Desert Hedgehog/Patched 1 signaling specifies fetal Leydig cell fate in testis organogenesis. Genes Dev. 2002;16(11):1433–1440. 12050120 10.1101/gad.981202 PMC186321

[63] Canto P, Söderlund D, Reyes E, Méndez JP Mutations in the *desert hedgehog* (*DHH*) gene in patients with 46,XY complete pure gonadal dysgenesis. J Clin Endocrinol Metab. 2004;89(9):4480–4483. 15356051 10.1210/jc.2004-0863

[64] Stévant I, Kühne F, Greenfield A, Chaboissier M-C, Dermitzakis ET, Nef S Dissecting cell lineage specification and sex fate determination in gonadal somatic cells using single-cell transcriptomics. Cell Reports. 2019;26(12):3272–3283.e3. 30893600 10.1016/j.celrep.2019.02.069

[65] Evilen da Silva T, Gomes NL, Lerário AM, Keegan CE, Nishi MY, Carvalho FM, Vilain E, Barseghyanm H, Martinez-Aguayo A, Forclaz MV, Papazian R, Pedroso de Paula LC, Costa EC, Carvalho LR, Jorge AA, Elias F, Mitchell R, Frade Costa EM, Mendonca BB, Domenice S Genetic evidence of the association of DEAH-box helicase 37 defects with 46,XY gonadal dysgenesis spectrum. J Clin Endocrinol Metab. 2019;104(12):5923–5934. 31287541 10.1210/jc.2019-00984

